# From rule-taking to rule-making chemistry: a minimal population–environment feedback model

**DOI:** 10.1098/rsif.2025.1306

**Published:** 2026-09-09

**Authors:** Celia Blanco

**Affiliations:** ^1^ Centro de Astrobiología (CSIC-INTA), Ctra de Torrejón a Ajalvir, km 4, Torrejón de Ardoz, Madrid, Spain; ^2^ Blue Marble Space, Seattle, WA, USA

**Keywords:** origins of life, nonlinear dynamics, population–environment systems

## Abstract

A key step towards life is the shift from chemistry that merely adapts to its surroundings to chemistry that actively reshapes the local physicochemical conditions that rule its own replication. Using a coupled population–environment feedback model, we identify the conditions under which environment-modifying activity, once present, can become self-sustaining in mixed chemical populations. When environmental conditions adjust instantaneously to population composition, environmental influence leaves no lasting trace and modifiers can spread only if initially common. By contrast, when environmental modification persists over the timescale of population change, production and decay generate heritable local conditions that give rise to bistability and alternative long-term outcomes. This persistence enlarges the basin leading to sustained environmental control, suggesting that even simple feedbacks could have allowed early chemical systems to move beyond passive chemistry and begin constructing the conditions that support their own propagation.

## 1. Introduction

The origin of life is often described as the emergence of chemical systems capable of sustaining organization, reproduction and adaptive change. Classical models illuminate different ways in which such organization might arise and persist. For example, autocatalytic sets show how reaction networks can collectively maintain and propagate themselves [1]; hypercycle models demonstrate how cooperating replicators can overcome parasitic alternatives once template-based heredity is present [2]; and protocell constructs illustrate how compartmentalization can couple metabolism, growth and division into integrated units [3]. Despite capturing essential features of early evolution, these frameworks typically focus on the dynamics of replicating entities themselves and treat reaction conditions as externally imposed and fixed, rather than shaped or stabilized by the chemical systems in which replication occurs.

However, life is distinguished by its capacity to modify the conditions under which it operates [4]. Niche construction [5] and ecological scaffolding [6] show that living systems shape aspects of their environment in ways that influence subsequent evolutionary dynamics. Origins of life research likewise suggests that early chemistries may have begun to stabilize favourable local conditions rather than merely endure externally imposed ones. In particular, recent work on autocatalytic chemical ecosystems emphasizes the continuity between ecological and evolutionary dynamics in open chemical systems, showing how feedback between reaction networks and their environments can support persistence and adaptive change prior to genetic inheritance [7,8]. Experimental support for this perspective comes from recent work on synthetic replicator ecosystems, in which self-replicating molecules modify their chemical environment in ways that feed back on replication dynamics, generating complex eco-evolutionary behaviour [9]. Theoretical studies of evolutionary games with environmental feedbacks have shown that such coupling generically produces bistability, tipping points and history-dependent outcomes even in simple strategic settings [10]. In prebiotic modelling, the metabolically coupled replicator systems (MCRS) framework demonstrates how replicators that invest in costly metabolic functions—thereby modifying their local chemical environment—can coexist and resist parasites on mineral surfaces [11,12]. More broadly, systematic surveys of autocatalytic cycles across element groups reveal that environment-modifying autocatalysis is chemically widespread, suggesting that the feedback motif formalized here may have been readily available in prebiotic settings [13].

This convergence suggests that two broad classes of constraints are central to the emergence of life. The first concerns reliable replication and information preservation, including fidelity, stability and protection of replicating structures. The second concerns the ability of chemical systems to modify or stabilize the conditions under which replication occurs. Much of the origin-of-life literature has focused on the former, developing detailed accounts of how inheritance and replication might become robust. By contrast, the latter has received comparatively less attention, particularly in pre-genetic contexts where regulation and environmental coupling must precede encoded heredity. This emphasis aligns with discontinuist perspectives, which argue that the transition from molecular evolution to biological organization is marked not by replication alone, but by the emergence of regulatory mechanisms that integrate system and environment [14]. Understanding when and how such endogenous feedback can first arise in chemical populations is therefore essential for explaining the transition from rule-taking to rule-making chemistry.

Here, we develop a minimal population–environment feedback model to examine when environment-modifying activity, once present, can become self-sustaining and reshape the conditions governing chemical propagation. We analyse the conditions under which such rule-making strategies can spread, how their fate depends on initial abundance and environmental state, and how even a single dynamical environmental variable reshapes the phase structure governing long-term outcomes.

## 2. Model

**Figure 1.**
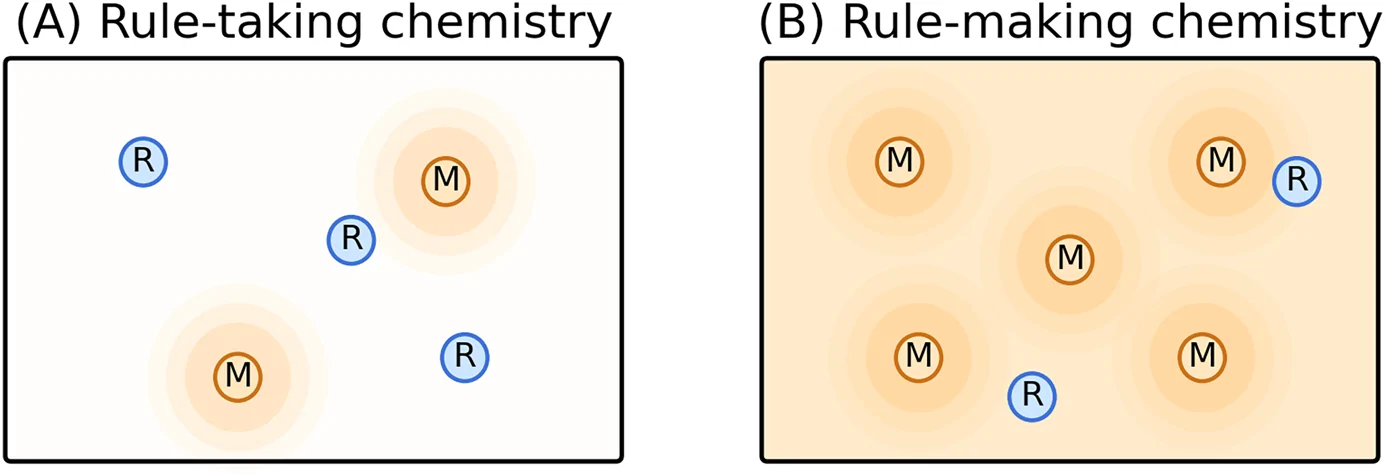
Conceptual illustration of the feedback model; rule-takers are denoted by R and rule-makers are denoted by M. (A) The environment remains tightly coupled to population composition, either because relaxation is fast or because rule-makers are rare, producing effectively instantaneous feedback. (B) Environmental modification accumulates when relaxation is slow or rule-makers are sufficiently abundant, allowing changes in environmental quality to persist over the timescale of population dynamics. Both panels represent different parameter regimes of the same underlying dynamical system.

We consider a well-mixed population composed of two types of chemical replicators: rule-takers (R), which reproduce under external physicochemical conditions they do not modify, and rule-makers (M), which incur a cost to alter those conditions (figure 1). Let y denote the frequency (proportion) of rule-makers in the population and 1−y the frequency of rule-takers, implicitly assuming that the total population is regulated on a faster timescale than changes in composition, as in chemostat-like or dilution-balanced settings. Such open-system conditions are natural idealizations of prebiotic environments with continuous material throughput (e.g. hydrothermal vents, flow-through porous media, tidal pools with periodic replenishment).

The local environment is represented by a scalar variable E, which acts as a coarse-grained descriptor of catalytic, buffering or stabilizing factors that influence replication rates. The variable E is shared by both types, with rule-makers increasing it while paying a cost, and rule-takers benefiting from it without contributing to its production. Rule-makers are thus the source of environmental change, and the resulting environmental state feeds back on replication.

Each chemical species replicates at a *per capita* rate, denoted by a fitness π, which determines its rate of increase. Rule-maker fitness includes three components—a baseline performance s relative to rule-takers, a cost c>0 for modifying the environment, and a feedback benefit pE that increases with environmental quality, where p>0 is a sensitivity parameter quantifying how strongly environmental quality enhances rule-maker replication and E is the environmental variable defined below. Thus,

(2.1) $$\pi_{M}=s-c+pE,\;\pi_{R}=1,$$

where the rule-taker's fitness is normalized to unity. Because the replicator equation (equation (2.2) below) depends only on the fitness difference $\pi_{M}-\pi_{R}$, any environmental benefit shared equally by both types cancels and does not affect the dynamics. The term pE in $\pi_{M}$ therefore represents the *differential* advantage that rule-makers derive from the environmental state; for example, because the modifier they produce is specifically complementary to their own catalytic requirements. The normalization $\pi_{R}=1$ is a standard convention in replicator dynamics that sets the scale of absolute fitness without loss of generality.

Under the replicator equation [15,16], the frequency of rule-makers changes according to

(2.2) $$\dot{y}=y(\pi_{M}-\bar{\pi }),$$

where the mean fitness is

(2.3) $$\bar{\pi }=y\pi_{M}+(1-y)\pi_{R}.$$

Substituting equations (2.1) and  (2.3) into equation (2.2) gives

(2.4) $$\dot{y}=y(1-y)(s-c+pE-1),$$

a quadratic equation in y for fixed E, whose coupling to the environmental dynamics (equation (2.5) below) produces an effective cubic dependence on y along the environmental nullcline and determines the qualitative behaviour of the system.

Environmental conditions evolve according to

(2.5) $$\dot{E}=\alpha y-\beta E,$$

where α quantifies the rate at which rule-makers improve the environment and β the rate at which environmental conditions relax back towards their abiotic baseline. The decay term βE represents background physicochemical processes that act independently of the replicating chemistry; environmental modification can therefore persist only when rule-maker production outpaces this decay.

## 3. Results

### 3.1. Stability analysis

The coupled population–environment system defined by equations (2.4) and (2.5) admits two natural boundary equilibria. The first ($A_{1}$) corresponds to extinction of rule-makers, so that the population is entirely rule-taking and the environment remains at its baseline level. The second ($A_{2}$) corresponds to full dominance of rule-makers, in which environmental quality is maintained at a steady state set by the balance between production and relaxation. In addition to these boundary states, an interior equilibrium (S) may exist in which rule-takers and rule-makers coexist at fixed frequencies together with a non-trivial environmental state (see appendix A). These three equilibria are given by

$$A_{1}=(0,0),\;A_{2}=(1,\alpha /\beta ),\;S=(y_{s},E_{s})=\left(\frac{p_{c}}{p_{\mathrm{eff}}},\;\frac{\alpha }{\beta }\frac{p_{c}}{p_{\mathrm{eff}}}\right).$$

The appearance of the interior equilibrium is governed by the threshold condition

$$p_{\mathrm{eff}}>p_{c}=c+1-s,$$

where c is the cost of environmental modification and 1−s is the baseline disadvantage of the modifying strategy, and

(3.1) $$p_{\mathrm{eff}}=p\;\frac{\alpha }{\beta }$$

is the effective strength of feedback relative to environmental decay.

For $p_{\mathrm{eff}}\leq p_{c}$, the only admissible equilibrium is the rule-taking state $A_{1}$, which is globally stable. For $p_{\mathrm{eff}}>p_{c}$, $A_{1}$ and $A_{2}$ are both stable, whereas S is a saddle whose stable manifold forms the separatrix in the (y,E) plane that divides the basins of attraction of the two boundary states (see appendix A).

**Figure 2.**
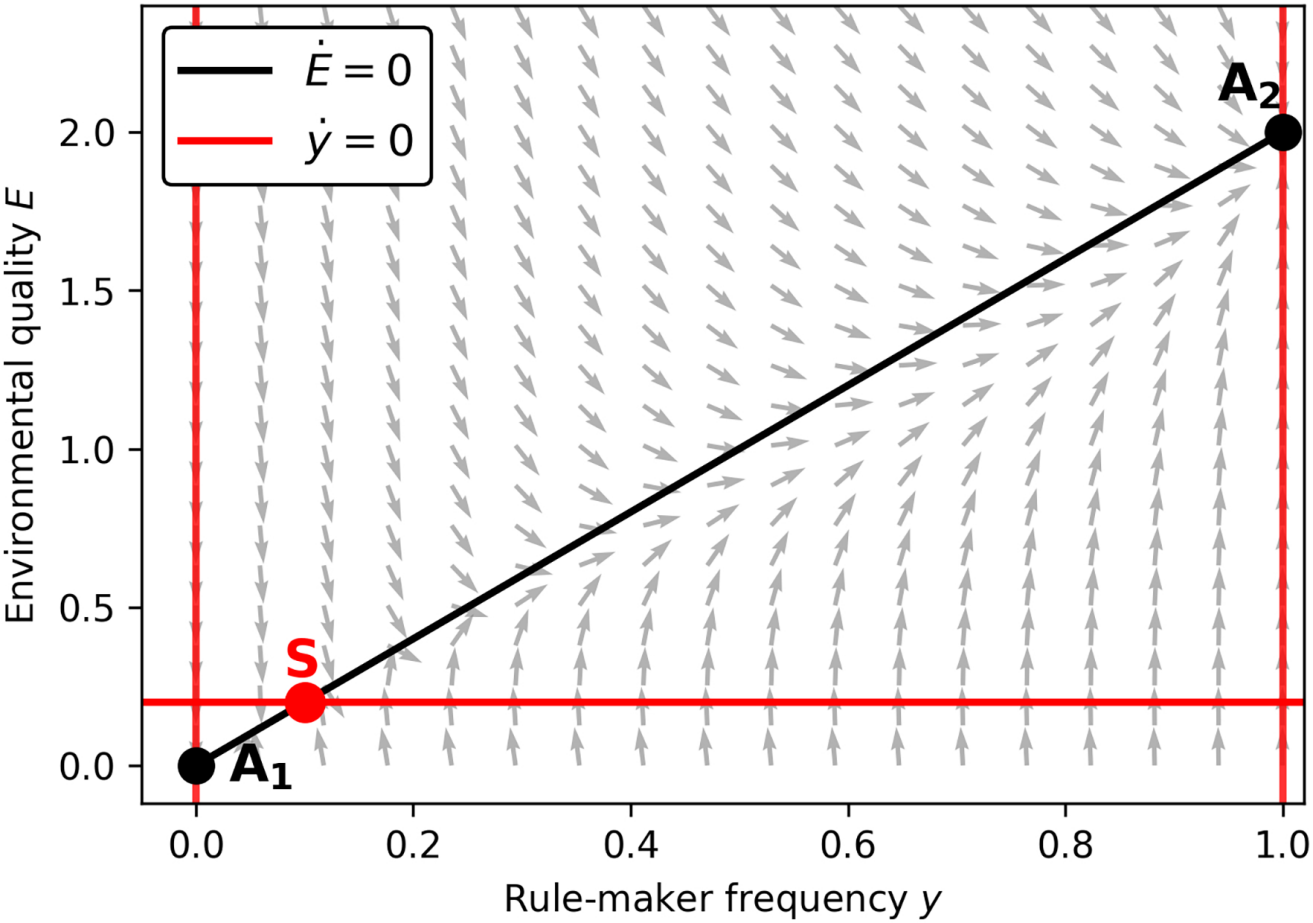
Nullcline structure and flow of the population–environment feedback model (figure 1). Black solid line: environmental nullcline $\dot{E}=0$ with E=(α/β)y. Red solid lines: population nullclines $\dot{y}=0$, including the interior nullcline E=(1−s+c)/p and the invariant boundaries y=0 and y=1. The fixed points are $A_{1}=(0,0)$, $A_{2}=(1,2.0)$, and the interior saddle $S=(y_{s},E_{s})=(0.1,0.2)$. Grey arrows indicate the normalized direction field. Parameters: s=1.0, c=0.2, p=1.0, α=1.0, β=0.5, giving $p_{c}=c+1-s=0.2$ and $p_{\mathrm{eff}}=p\alpha /\beta =2.0$.

The geometry of these coupled dynamics can be visualized through the nullclines and direction field of the (y,E) system, which illustrate how trajectories are guided through phase space (figure 2). The vector field points towards the environmental nullcline $\dot{E}=0$ from both above and below, indicating that it acts as an attracting manifold in the vertical direction. Once close to this curve, further evolution is dominated by changes in the population composition y. In other words, deviations of the environmental modifier from its production–decay balance relax automatically, after which the remaining evolution reflects changes in the relative abundance of rule-makers and rule-takers. This two-dimensional population–environment system, in which environmental modification persists over the same timescale as population change, defines what we refer to as the dynamic-environment regime.

**Figure 3.**
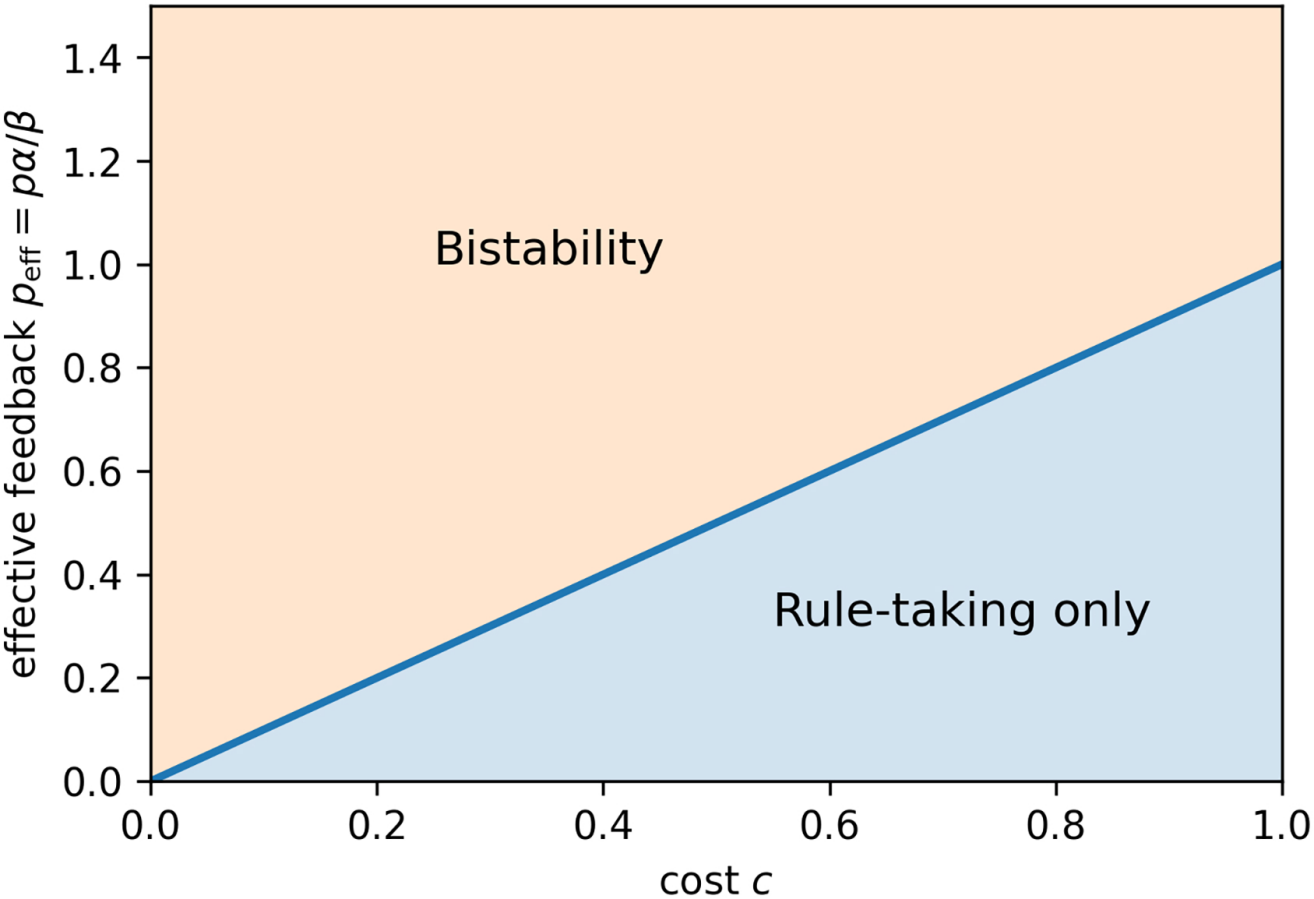
Binary phase diagram of dynamical regimes as a function of cost c and effective feedback strength $p_{\mathrm{eff}}=p\alpha /\beta$ for s=1. At and below the threshold line $p_{\mathrm{eff}}=p_{c}$, only the rule-taking equilibrium $A_{1}$ exists and is globally stable. Above the line, the system is bistable, with coexistence of the rule-taking equilibrium $A_{1}$ and the rule-making equilibrium $A_{2}$ separated by the interior saddle S.

Within the bistable region of the dynamic-environment regime, the two boundary attractors are separated in phase space by a curved separatrix in the (y,E) plane, whose geometry determines how initial conditions are partitioned between rule-taking and rule-making outcomes. The boundary between these regimes is linear in $\left(c,p_{\mathrm{eff}}\right)$ for fixed s (figure 3), reflecting the analytic condition for the appearance of the interior saddle. This linear frontier encodes a direct trade-off between cost and feedback strength. Increasing the energetic or functional cost of rule-making can be compensated by proportionally stronger environmental feedback, thereby preserving bistability.

**Figure 4.**
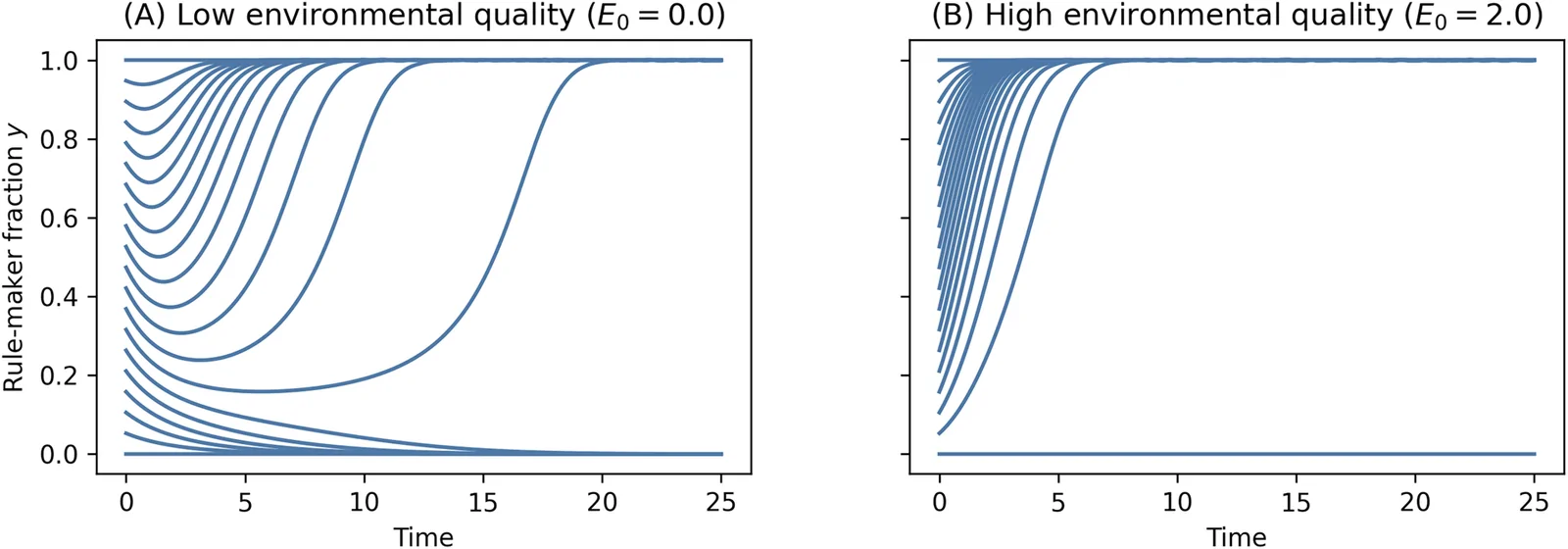
Time evolution of rule-maker frequency y(t) for a uniform range of initial population compositions $y_{0}$ under two different initial environmental conditions within the bistable regime. (A) Low initial environmental quality (modifier concentration) ($E_{0}=0$). (B) High initial environmental quality ($E_{0}=2$). Parameters: s=1.0, c=0.5, p=0.8, α=1.0, β=0.2, giving $p_{c}=c+1-s=0.5$ and $p_{\mathrm{eff}}=p\alpha /\beta =4.0$. For low $E_{0}$, only sufficiently large initial rule-maker fractions overcome decay and approach the rule-making attractor, whereas for high $E_{0}$ even initially rare rule-makers reliably invade.

Environmental history strongly shapes long-term population outcomes within the bistable regime. Figure 4 shows trajectories of the coupled system across a uniform range of initial rule-maker frequencies $y_{0}$ for two contrasting initial environmental states. When the initial environment is poor ($E_{0}=0$), only sufficiently large initial rule-maker fractions cross the separatrix and approach the rule-making attractor, while smaller populations decay towards the rule-taking state. By contrast, when the environment is initially favourable ($E_{0}=2$), even rare rule-makers reliably grow and invade, and almost all trajectories converge to the rule-making attractor. This demonstrates that pre-existing environmental conditioning can drastically alter the basin structure, lowering the threshold for rule-makers to establish themselves. The same feedback parameters therefore support radically different evolutionary outcomes depending solely on the environmental state from which the system is initialized.

### 3.2. Quasi-steady environment limit

To isolate the role of environmental persistence, it is instructive to consider the limit in which environmental dynamics are slaved to population composition. In this quasi-steady environment limit, deviations of the environmental variable relax rapidly compared to changes in population composition, so that the system remains close to the environmental nullcline $\dot{E}=0$ throughout its evolution. The environmental state is therefore no longer an independent dynamical degree of freedom but is approximately determined by the instantaneous rule-maker frequency according to

$$E\approx \frac{\alpha }{\beta }\;y.$$

Substituting this quasi-static relation into the population equation (2.4) reduces the coupled two-dimensional dynamics to an effective one-dimensional replicator equation with instantaneous frequency-dependent feedback:

$$\dot{y}=y(1-y)(s-c+p_{\mathrm{eff}}\;y-1),$$

where $p_{\mathrm{eff}}=p\alpha /\beta$ is the effective feedback strength defined in equation (3.1).

The reduced dynamics admits equilibria at the same values of the rule-maker fraction y as in the full system: a rule-taking state at y=0, a rule-making state at y=1, and an interior equilibrium at $y=p_{c}/p_{\mathrm{eff}}$ when $p_{\mathrm{eff}}>p_{c}=c+1-s$. However, the phase-space geometry is fundamentally altered. In the quasi-steady environment limit, the curved separatrix present in the dynamic-environment case collapses onto a single, sharp threshold at $y=y_{s}=p_{c}/p_{\mathrm{eff}}$, so that long-term outcomes depend exclusively on the initial rule-maker frequency. Trajectories with $y_{0}<y_{s}$ converge to the rule-taking state, whereas those with $y_{0}>y_{s}$ converge to the rule-making state, independent of the initial environmental condition.

**Figure 5.**
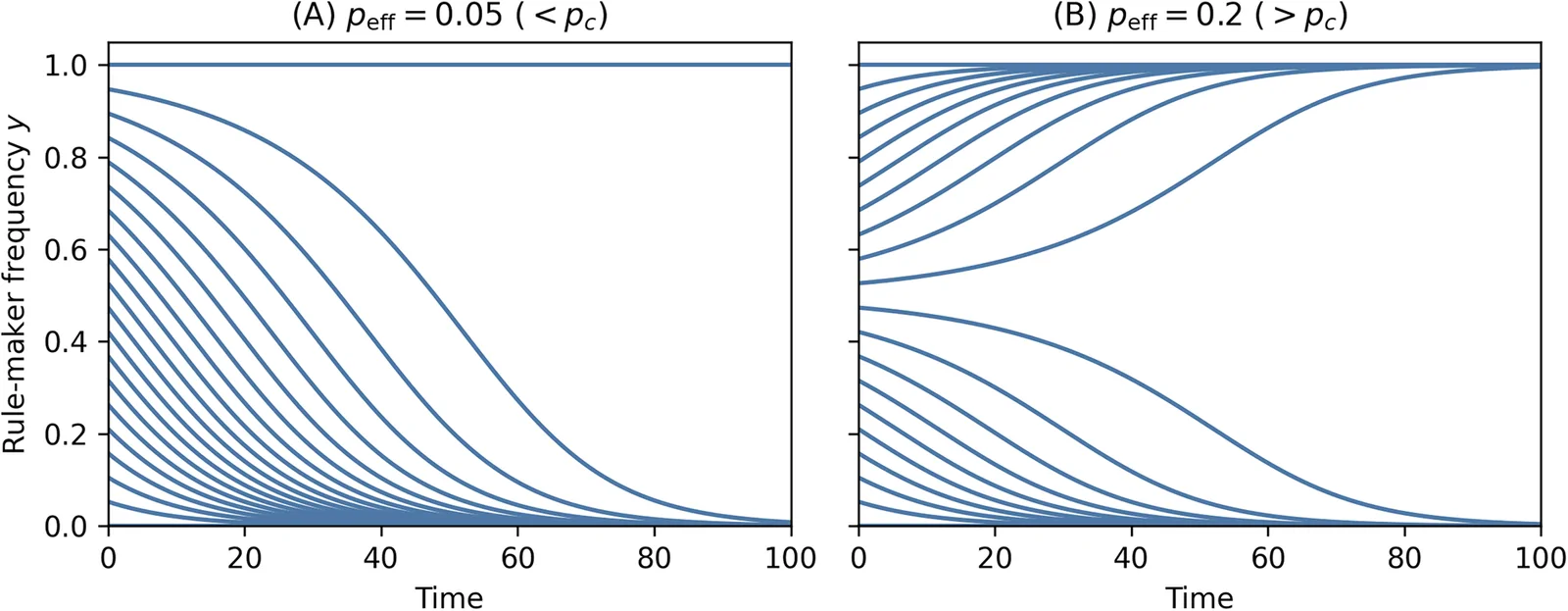
Dynamics in the quasi-steady environment limit. Time evolution of the rule-maker frequency y(t) for a uniform range of initial conditions $y_{0}$ under the one-dimensional reduced dynamics obtained by eliminating the environmental variable via the quasi-steady relation E≈(α/β)y. (A) Subcritical effective feedback ($p_{\mathrm{eff}}<p_{c}$), for which all trajectories converge to the rule-taking state y=0. (B) Supercritical effective feedback ($p_{\mathrm{eff}}>p_{c}$), for which a sharp threshold at $y=y_{s}=p_{c}/p_{\mathrm{eff}}$ separates trajectories that decay towards the rule-taking state from those that converge to the rule-making state y=1. Parameters: s=1.0, c=0.1, with $p_{c}=c+1-s=0.1$.

The consequences of this reduction are illustrated in figure 5, which shows trajectories of the reduced one-dimensional dynamics across a uniform range of initial rule-maker frequencies for effective feedback strengths below and above the threshold. For subcritical effective feedback ($p_{\mathrm{eff}}<p_{c}$), all trajectories relax to the rule-taking state. For supercritical effective feedback ($p_{\mathrm{eff}}>p_{c}$), a sharp threshold separates trajectories that decay from those that grow towards the rule-making state. Unlike in the dynamic-environment regime analysed above, environmental history plays no role in this limit; only the initial population composition determines the outcome.

The purpose of this reduction is not to derive a surprising result in isolation, but to establish a baseline against which the dynamic-environment regime can be compared. In the full two-dimensional system (§3.1), the environmental variable retains memory of past population states, so that both the initial composition $y_{0}$ and the initial environmental quality $E_{0}$ jointly determine outcomes. This history dependence—absent in the quasi-steady limit—is the qualitatively new feature introduced by environmental persistence, and it is what allows pre-existing environmental conditions to enlarge the basin of attraction for rule-making chemistry (cf. figure 4).

### 3.3. Minimal chemical realization of rule-making feedback

The population–environment feedback model admits a direct realization as a minimal chemical reaction network. This construction clarifies how the abstract environmental variable E corresponds to a physically meaningful modifier species and shows that the feedback structure encoded in equations (2.4) and (2.5) arises naturally from standard mass-action kinetics under well-defined assumptions.

We consider five chemical species: a rule-taking replicator R, a rule-making replicator M, a generic substrate S supplying material or free energy for replication, a chemical precursor F, and an environmental modifier B. The modifier B represents any chemical factor produced by M that enhances the local replication rate of M, for example through catalysis, buffering, metal chelation or stabilization of reaction conditions. The reaction network is defined by the following elementary processes:

(3.2)R+S→kR2R,(3.3)M+S→kM(1−γ)2M,(3.4)M+S+B→kfb2M+B,(3.5)M+F→αM+B,(3.6)R→μR∅,(3.7)M→μM∅,(3.8)B→β∅.

Here, equations (3.2) and (3.3) describe baseline replication of R and M from a common substrate S; the rule-maker replication rate is reduced by the factor (1−γ), where γ∈(0,1) represents the allocation of a fraction of M's catalytic capacity to modifier production rather than self-replication. Equation (3.4) represents enhanced, feedback-mediated replication of M in the presence of the modifier B, which acts catalytically and is regenerated in the process. Equation (3.5) describes production of the environmental modifier by M from the precursor F (e.g. catalytic cofactors, metal-chelating ligands or buffering compounds). Equations (3.6)–(3.8) describe first-order loss of each species owing to degradation, dilution or outflow.

We assume that both S and F are maintained at approximately constant concentrations $S_{0}$ and $F_{0}$, respectively, by external supply, as in a chemostat or continuously driven prebiotic reactor. Under these assumptions, the mass-action dynamics for the concentrations R(t), M(t) and B(t) take the form

(3.9)dRdt=(kRS0−μR)R,(3.10)dMdt=(kM(1−γ)S0−μM+kfbS0B)M,(3.11)dBdt=αM−βB.

This assumption implies regulation of total replicator abundance on a faster timescale than changes in population composition, consistent with the frequency-based formulation used above.

The reaction scheme induces *per capita* growth rates

$$\pi_{R}=k_{R}S_{0}-\mu_{R},\;\pi_{M}=k_{M}\left(1-\gamma \right)S_{0}-\mu_{M}+k_{\mathrm{fb}}S_{0}B,$$

which determine exponential replication rates and correspond directly to the growth coefficients used in the population-level description. Writing the dynamics in terms of the rule-maker frequency y=M/(R+M) yields the standard replicator form

$$\dot{y}=y(1-y)(\pi_{M}-\pi_{R}).$$

Grouping parameters shows that the intrinsic advantage of rule-makers corresponds to $s-c=k_{M}\left(1-\gamma \right)S_{0}-\mu_{M}$, while the strength of environmental feedback corresponds to $p=k_{\mathrm{fb}}S_{0}$, with the environmental variable identified chemically as E≡B. Under these identifications, the chemical model reduces exactly to equation (2.4) (see appendix B).

**Figure 6.**
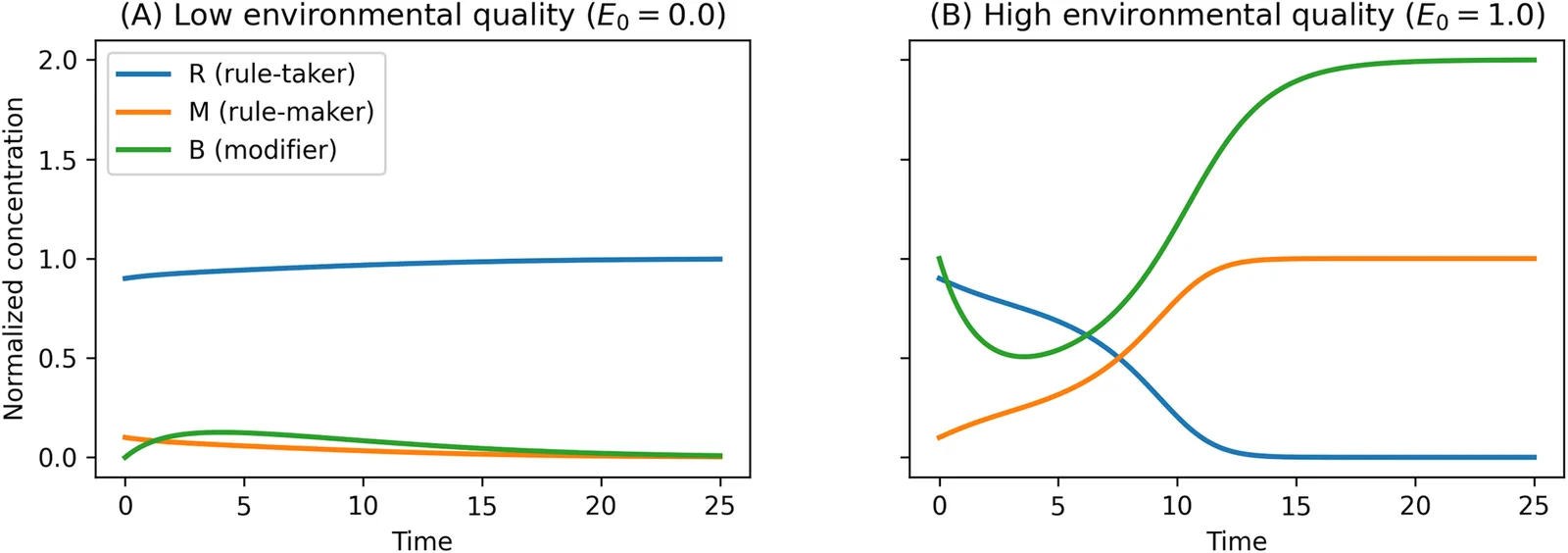
Dynamics of a chemically interpreted population–environment feedback model. Time series of normalized concentrations of the rule-taker R (blue), rule-maker M (orange) and modifier B (green) for the bistable parameter set s=1.0, c=0.2, p=0.8, α=1.0 and β=0.5, so that $p_{c}=c+1-s=0.2$ and $p_{\mathrm{eff}}=p\alpha /\beta =1.6$. In both panels the initial rule-maker fraction is $y_{0}=0.1$; the two panels differ only in the initial environmental quality. (A) Low initial environmental quality ($E_{0}=0$) leads to extinction of rule-makers and decay of the modifier concentration. (B) High initial environmental quality ($E_{0}=1.0$) allows rule-makers to increase in abundance, maintain a positive modifier concentration, and drive the system to the rule-making attractor.

Figure 6 shows the feedback dynamics expressed as normalized concentrations of rule-takers, rule-makers and modifier species, obtained by integrating equations (2.4) and (2.5). For bistable parameters, these trajectories illustrate the same regime structure identified at the population level. When the initial modifier concentration is low, modifier decay prevents sustained feedback and the system relaxes to the rule-taking state. When the modifier concentration is initially high, feedback between M and B is maintained, allowing rule-makers to increase in abundance and stabilize a persistent environmental state.

In this chemically grounded formulation, the environmental variable E corresponds to the concentration of a modifier species that is produced by rule-makers at a cost, enhances their own replication and decays on a characteristic timescale $\beta^{-1}$. The effective feedback strength $p_{\mathrm{eff}}=p\alpha /\beta$ therefore admits a direct chemical interpretation as the ratio between the catalytic enhancement rate of modifier-assisted replication and the lifetime of the modifier in the environment.

This minimal reaction network shows explicitly how population–environment feedback can arise from chemically plausible processes without invoking pre-existing genetic information or complex metabolic organization. Environmental modification persists only when supported by sufficient population abundance, reproducing the same bistable transition between externally constrained and self-stabilizing regimes identified in the population-level analysis.

## 4. Discussion

The results identify a sharp threshold separating qualitatively distinct regimes of chemical organization, consistent with the broader role of thresholds as organizing principles in origin-of-life scenarios. In many prebiotic contexts, small and continuous changes in parameters can produce discontinuous changes in system-level behaviour, marking transitions between regimes with fundamentally different modes of persistence and control [17]. In the present model, the relevant threshold concerns the persistence of population–environment feedback. Below a critical strength, environmental modification remains transient and does not alter long-term outcomes. Above it, modifier-producing populations can collectively stabilize the conditions that support their own persistence.

From a chemical perspective, this threshold reflects a balance between production and dissipation of environmentally active species. The environmental variable represents the concentration of a modifier produced by rule-makers and removed by background processes such as dilution, degradation or buffering. It is worth clarifying that E represents local physicochemical conditions on the timescale of replication rather than geological-scale landscape changes or the broader accumulation of chemical diversity; the latter enter the model only implicitly through β, which sets the rate at which the local environment relaxes back towards its abiotic baseline. When production exceeds dissipation, the modifier accumulates and reshapes the effective growth conditions experienced by the population. When dissipation dominates, environmental improvements fail to persist. The transition therefore marks a shift from environments that merely respond to chemistry to environments that are actively stabilized by it.

Importantly, this transition does not concern the fidelity of replication, the closure of reaction networks, or the stability of specific molecular structures. Instead, it concerns the stability of environmental influence itself. In threshold-based frameworks for the origin of life, this corresponds to a system–environment coupled transition, in which persistence depends jointly on internal population dynamics and environmental timescales [17]. The emergence of rule-making chemistry thus represents a qualitative change in how chemical systems relate to their surroundings, from passive dependence to partial environmental control. This interpretation becomes explicit in the chemically grounded formulation of the model. There, the environmental variable corresponds to the concentration of a concrete modifier species produced by rule-makers and removed by background processes. The effective feedback strength reflects the balance between how strongly the modifier enhances replication and how long it persists in the environment. When production outpaces loss, the modifier accumulates to levels that feed back to enhance the replication of its producers. When loss dominates, environmental effects remain short-lived. The minimal reaction network therefore provides a concrete chemical mechanism by which replicators can create, maintain and transmit enabling environmental conditions without requiring genetic encoding or complex metabolism.

Recent work has shown that the emergence of Darwin-like evolutionary dynamics may itself require crossing a threshold separating degradation-dominated chemistry from sustained propagation [18]. In that work, cooperative autocatalysts are shown to overcome such a barrier and enter a growth-dominated regime in which evolutionary invasion becomes possible. Related perspectives argue more broadly that ecological and evolutionary dynamics form a continuum even in purely chemical systems, with selection acting on persistence and dispersal prior to genetic encoding [7]. The transition identified here is conceptually distinct. Replication is assumed to be already possible, but the results show that replicating populations may nevertheless remain confined to a rule-taking regime unless population–environment feedback persists strongly enough to stabilize favourable conditions. In this sense, rule-making chemistry represents a subsequent threshold, marking the shift from propagation under given conditions to partial control over the conditions that govern propagation itself.

The model also connects naturally to niche-construction and constraint-inheritance frameworks [5]. Modifier-producing replicators generate environmental conditions that persist long enough to influence the selective environment of subsequent generations, providing a minimal example of non-genetic inheritance. Once established, these conditions stabilize the modifier-dominated regime, much as niche construction stabilizes organism–environment couplings in biological systems. In this sense, the model illustrates how chemical populations can acquire a rudimentary form of autonomy by reinforcing the constraints that favour them.

Recent experimental work on synthetic replicator ecosystems provides a concrete chemical precedent for the feedback mechanism modelled here. Liu *et al.* demonstrated that self-replicating molecules can alter the composition of their chemical environment through light-driven redox processes, and that these changes feed back on replicator fitness, driving transitions between alternative community states [9]. The present model provides a theoretical framework for understanding such transitions in terms of feedback strength and environmental persistence. More broadly, game-theoretic models with environmental feedback have shown that strategy–environment coupling generically produces the same qualitative features—bistability, hysteresis and tipping points—identified here [10], while the MCRS framework illustrates how costly metabolic investment by replicators can be sustained through spatial structure and local environmental modification [11,12]. The widespread occurrence of autocatalytic cycles across diverse element groups [13] further suggests that the chemical prerequisites for environment-modifying feedback are not exotic but may have been broadly available under prebiotic conditions.

A central consequence of the threshold is strong history dependence. Initial conditions jointly determine whether the system crosses the separatrix into the modifier-dominated state or relaxes to the rule-taking state. Such sensitivity to initial conditions may have been important on the early Earth, where heterogeneous microenvironments would have intermittently supported the accumulation of environmental modifiers. Only locales that crossed the threshold would have supported self-maintaining chemical organization, potentially seeding the emergence of more complex evolutionary dynamics. At the same time, the model clarifies why such transitions should be rare rather than inevitable. Environmental modification is widespread in chemistry, but most modifications are too weak, too transient or too costly to persist long enough to reshape selection. In the present framework, modifiers that decay rapidly are erased before they can influence population composition, while modifiers that enhance replication only weakly fail to offset their production costs. Even when bistability exists, successful transition into the modifier-dominated state requires crossing a separatrix that depends jointly on population composition and environmental state. The model therefore does not imply that feedback generically produces autonomy. Instead, it shows that autonomy emerges only under restricted conditions of sufficient feedback strength, persistence and favourable history.

Despite its simplicity, the model suggests qualitative experimental predictions. In laboratory reactors or serial-dilution experiments, one could compare a resource-consuming replicator with a variant that also produces a stabilizing modifier. The theory predicts bistability when environmental modification is strong relative to cost, with hysteresis under controlled perturbations providing evidence for the predicted feedback threshold. More generally, the framework highlights population–environment feedback as a distinct axis along which chemical systems can transition from passive adaptation to partial environmental control. From a mechanistic perspective, the abstract parameters of the model—particularly the feedback sensitivity p, the modifier production rate α and the cost c—are ultimately determined by the underlying chemical reaction mechanisms. Classical mechanistic analysis, as pioneered by Ingold and others [19], provides a framework for relating macroscopic rate parameters to elementary bond-making and bond-breaking steps. Connecting the coarse-grained parameters of the present model to detailed mechanistic descriptions of specific prebiotic reactions is an important direction for grounding the feedback threshold in concrete chemistry.

An important complementary perspective concerns the molecular complexity of the chemical species involved. Measures of molecular complexity [20–23], functional information frameworks [24,25] and prebiotic scenario models that foreground the role of chemical complexity [26,27] offer quantitative tools for characterizing the threshold between passive and environment-modifying chemistry. In the present model, the transition to rule-making behaviour depends on the existence of replicators capable of producing an environmental modifier at sufficient rate and specificity. The molecular complexity required for such function is not addressed by our minimal model, which treats the modifier-production capability as a given parameter. Connecting complexity-theoretic measures to the feedback parameters identified here—for instance, asking what minimum molecular complexity is required for a replicator to achieve a given modifier-production rate α or feedback sensitivity p—is an important direction for future work that could help locate the sharp threshold identified here within a broader landscape of chemical complexity. Indeed, molecular complexity has already been used to date stages of prebiotic chemical evolution [22] and to show that complexity constrained the early recruitment of amino acids into the genetic code [23,28]. Extending this approach to environment-modifying chemistry is a natural next step.

In summary, the model isolates a key ingredient in the emergence of autonomy. Early evolutionary dynamics may have depended not only on replication with variation and selection, but also on the capacity of chemical systems to stabilize the constraints enabling their own persistence. The results suggest that the transition from rule-taking to rule-making chemistry is neither inevitable nor ubiquitous, but rare and conditional, arising only when feedback is strong enough to persist across the timescales that shape population change.

## Data Availability

Data and relevant code for this research work are stored in GitHub: http://github.com/celiablanco/RuleMaking and have been archived within the Zenodo repository [29].

## Appendix A: Fixed-point and stability analysis

The fixed points for the coupled system defined by equations (2.4) and (2.5) satisfy $\dot{y}=0$ and $\dot{E}=0$ simultaneously. From $\dot{E}=0$ we obtain the environmental nullcline

(A.1) $$E=\frac{\alpha }{\beta }\;y.$$

Substituting equation (A 1) into $\dot{y}=0$ yields the three equilibria

(A.2) $$(y_{0},E_{0})=(0,0),\;(y_{1},E_{1})=(1,\alpha /\beta ),\;(y^{*},E^{*})=\left(\frac{p_{c}}{p_{\mathrm{eff}}},\;\frac{\alpha }{\beta }\frac{p_{c}}{p_{\mathrm{eff}}}\right),$$

where

(A.3) $$p_{c}=c+1-s,\;p_{\mathrm{eff}}=\frac{p\alpha }{\beta }.$$

The interior equilibrium exists in the admissible region only when $0<y^{*}<1$, or what is the same, when $p_{\mathrm{eff}}>p_{c}$.

Linear stability is determined from the Jacobian of the two-dimensional flow:

(A.4) $$J(y,E)=\left(\begin{array}{cc} (1-2y)(s-c+pE-1) & p\;y(1-y)\\ \alpha & -\beta \end{array}\right).$$

Evaluating equation (A 4) at the boundary equilibria in equation (A 2) and using equation (A 3), gives

(A.5) $$J(0,0)=\left(\begin{array}{cc} -p_{c} & 0\\ \alpha & -\beta \end{array}\right),\;J(1,\alpha /\beta )=\left(\begin{array}{cc} p_{c}-p_{\mathrm{eff}} & 0\\ \alpha & -\beta \end{array}\right).$$

Hence $\left(y_{0},E_{0})=(0,0\right)$ is stable whenever $p_{c}>0$, and $\left(y_{1},E_{1})=(1,\alpha /\beta \right)$ is stable when $p_{\mathrm{eff}}>p_{c}$.

At the interior equilibrium $\left(y^{*},E^{*}\right)$, direct evaluation of the trace and determinant shows that

$$\det J(y^{*},E^{*})=-\alpha \;p\;y^{*}(1-y^{*})<0,$$

whenever it exists. The determinant is therefore always negative in the admissible regime, implying one positive and one negative eigenvalue and confirming that $\left(y^{*},E^{*}\right)$ is a saddle point.

## Appendix B: Derivation of the reduced replicator–environment system

The deterministic mass-action equations for the three variables are

(B.1)dRdt=(kRS0−μR)R,(B.2)dMdt=(kM(1−γ)S0−μM+kfbS0B)M,(B.3)dBdt=αM−βB.

The coefficients in front of R and M define the *per capita* growth rates:

$$\pi_{R}=k_{R}S_{0}-\mu_{R},\;\pi_{M}=k_{M}\left(1-\gamma \right)S_{0}-\mu_{M}+k_{\mathrm{fb}}S_{0}B.$$

Let N=R+M and y=M/N. Differentiating y yields

$$\dot{y}=\frac{N\;\dot{M}-M\;\dot{N}}{N^{2}}.$$

Using $\dot{R}=\pi_{R}R$ and $\dot{M}=\pi_{M}M$, we obtain

$$\dot{N}=\pi_{R}R+\pi_{M}M.$$

Substituting into the expression for $\dot{y}$ and simplifying gives the standard replicator form:

$$\dot{y}=y(1-y)(\pi_{M}-\pi_{R}).$$

To connect this with the reduced notation in the main text, we rescale time so that $\pi_{R}=1$ and define the parameter combinations

$$s-c=k_{M}\left(1-\gamma \right)S_{0}-\mu_{M},\;p=k_{\mathrm{fb}}S_{0}.$$

Under this rescaling,

$$\dot{y}=y(1-y)\;(s-c+pB-1),$$

which matches the functional form of equation (2.4) once we identify the environmental variable as E≡B.

The environmental dynamics follow directly from equation (B 3). Under chemostat-like conditions the total abundance N remains approximately constant, allowing M=yN and therefore

$$\dot{E}=\alpha \;yN-\beta E=\tilde{\alpha }\;y-\beta E,$$

where $\tilde{\alpha }=\alpha N$ is absorbed into the effective production rate. This reproduces the environmental equation used in the main text.
